# Valuing the cost of improving Chilean primary vaccination: a cost minimization analysis of a hexavalent vaccine

**DOI:** 10.1186/s12913-020-05115-7

**Published:** 2020-04-09

**Authors:** Ignacio Olivera, Carlos Grau, Hugo Dibarboure, Juan Pablo Torres, Gustavo Mieres, Luis Lazarov, Fabián P. Alvarez, Juan Guillermo López Yescas

**Affiliations:** 1 Centro de Investigaciones Económicas, CINVE, Salud, Montevideo, Uruguay; 2 Sanofi Pasteur, Montevideo, Uruguay; 3 Chile Departamento de Pediatría y Cirugía Infantil Oriente, Facultad de Medicina, Universidad de Chile, Santiago, Chile; 4 Sanofi Pasteur, Lyon, France; 5 Sanofi, Avenida Universidad 1738, Coyoacan, 04000 Mexico City, Mexico

**Keywords:** Vaccination, Cost, Pediatric, Polio

## Abstract

**Background:**

The phased withdrawal of oral polio vaccine (OPV) and the introduction of inactivated poliovirus vaccine (IPV) is central to the polio ‘end-game’ strategy.

**Methods:**

We analyzed the cost implications in Chile of a switch from the vaccination scheme consisting of a pentavalent vaccine with whole-cell pertussis component (wP) plus IPV/OPV vaccines to a scheme with a hexavalent vaccine with acellular pertussis component (aP) and IPV (Hexaxim®) from a societal perspective. Cost data were collected from a variety of sources including national estimates and previous vaccine studies. All costs were expressed in 2017 prices (US$ 1.00 = $Ch 666.26).

**Results:**

The overall costs associated with the vaccination scheme (4 doses of pentavalent vaccine plus 1 dose IPV and 3 doses OPV) from a societal perspective was estimated to be US$ 12.70 million, of which US$ 8.84 million were associated with the management of adverse events related to wP. In comparison, the cost associated with the 4-dose scheme with a hexavalent vaccine (based upon the PAHO reference price) was US$ 19.76 million. The cost of switching to the hexavalent vaccine would be an additional US$ 6.45 million. Overall, depending on the scenario, the costs of switching to the hexavalent scheme would range from an additional US$ 2.62 million to US$ 6.45 million compared with the current vaccination scheme.

**Conclusions:**

The switch to the hexavalent vaccine schedule in Chile would lead to additional acquisition costs, which would be partially offset by improved logistics, and a reduction in adverse events associated with the current vaccines.

## Background

Vaccines and associated pediatric immunization programs have been a vital public health intervention in reducing morbidity and mortality associated with many communicable diseases worldwide. These interventions not only protect those immunized, but also provide community-wide protection by reducing the spread of disease. In addition, the reduction in associated disease burden promotes healthy well-being which supports economic growth and poverty reduction [1, 2]. Improving vaccination coverage rates of pneumococcal, rotavirus, pertussis, measles, *Haemophilus influenzae* type b (Hib) and malaria (assuming their introduction in malaria-endemic countries) vaccines to 90% in seventy-two of the world’s poorest countries between 2011 and 2020 would save the lives of an estimated 6.4 million children aged < 5 years, representing $231 billion in the value of statistical lives saved [3].

Poliomyelitis is an acute paralytic disease caused by three poliovirus serotypes [4, 5]. The use of the formalin-inactivated Salk polio vaccine (IPV) (first introduced in 1955) and the Sabin oral polio vaccine (OPV) (introduced in the early 1960’s) in routine immunization programs and supplemental mass vaccination campaigns has led to the elimination or near elimination of poliomyelitis and polioviruses circulation from many countries [6]. Nonetheless, it is estimated that there are 10–20 million individuals worldwide living with poliomyelitis-related disabilities [7], though very little is known about the socio-economic consequences and healthcare costs of the disabilities.

OPV has been the vaccine of choice for the Global Polio Eradication Initiative (GPEI) of the World Health Organization (WHO) because of its low cost, ease of administration and superiority in inducing mucosal immunity [8, 9]. However, OPV vaccination carries some risks, namely the appearance of vaccine-associated paralytic poliomyelitis cases (VAPP) and the emergence of vaccine derived polioviruses (VDPV) [10]. The strategy for the elimination of all polioviruses, referred to as the polio endgame, includes two steps synchronized around the world for all countries still vaccinating with OPV. First the switch from trivalent OPV to bivalent OPV (as wild type OPV-2 no longer circulates), and second the switch from OPV to IPV in routine childhood immunization programs in order to avoid OPV-derived cases [10–12].

Vaccination against polio in Chile began in 1961 [13], and by 1975 the last case of wild polio was detected, 19 years before the South America region was declared polio-free [14]. As part of the Polio Eradication and Endgame Strategic Plan developed by the GPEI, the World Health Organization Strategic Advisory Group of Experts (WHO-SAGE) has recommended as a first step that all countries introduce at least one dose of IPV into their routine immunization programs [15], before the complete switch to immunization with IPV [16].

Combination vaccines have been developed and introduced in routine childhood immunization programs, allowing individuals to be simultaneously vaccinated against multiple infectious diseases including diphtheria, tetanus, pertussis, poliomyelitis, Hib and hepatitis B (HepB) [17, 18]. Combination vaccines have the additional advantages of increased vaccination coverage rates [19], improved vaccination timeliness, and reduction of the number injections required in an increasingly crowded immunization schedule [20–22].

Chile introduced a combined bivalent vaccine for diphtheria and pertussis (DP) into the routine childhood immunization program in 1950, which was later replaced by the trivalent vaccine that included pertussis (DTwP) in 1975. The tetravalent vaccine including Hib (DTwP-Hib) was introduced in 1996 and the switch to the pentavalent vaccine including HepB (DTwP-Hib-HepB) occurred in 2006. In 2015, Chile started to replace the 2-month OPV dose (used in combination with the pentavalent vaccine) with IPV in line with WHO-SAGE recommendations to support the polio eradication endgame strategy, with the vaccination series completed with OPV (used in combination with the pentavalent vaccine) administered at 4, 6 and 18 months. Then in February 2018, the hexavalent vaccine including IPV (DTaP-IPV-HepB-Hib) was introduced at 2 and 4 month and the vaccination series completed with the pentavalent (DTwP-Hib-HepB) vaccine and OPV both administered at 6 and 18 months [23]. More recently, in December 2018, the hexavalent (DTaP-IPV-HepB-Hib) vaccine was recommended to provide all four polio vaccine doses at 2, 4, 6 and 18 months [24].

In Latin America, there is an increasing role for economic evaluations in supporting evidence-based decision making. This has also applied to the assessment of new vaccines or immunization programs (see for example Glassman et al. 2016 [25], and the Pan-American Health Organization (PAHO) [26]). Most of these studies are based on cost-effectiveness and/or cost-utility analysis.

We undertook this study to assess the economic impact of moving from the Chilean vaccination scheme in 2016 to a new program based upon the use of a full hexavalent (DTaP-IPV-HepB-Hib) vaccine scheme at 2, 4, 6 and 18 month of age. Because the new scheme represents a replacement of antigens, for both OPV to IPV and wP to aP, without any significant difference in the efficacy of the switched antigens, we considered a cost-minimization analysis as the most appropriate economic evaluation methodology to utilize.

## Methods

The objective of the current study was to describe and analyze the cost differences between switching from the current vaccination scheme to a new scheme, based on a fully-liquid ready-to-use hexavalent vaccine. We considered a full switch between schemes to the new hexavalent program comprising 4 doses (three primary and one ‘booster’ doses). The programs were analyzed from the societal perspective.

The study cohort (infants eligible for vaccination) was taken from national statistics produced by the Chilean government [27]. The vaccination scheme in 2016, which consists of 4 doses of wP-containing pentavalent vaccine (DTwP-Hib-HepB) plus 1 dose IPV and 3 doses OPV, was based on the program from the Ministry of Health [23, 28] and the new scheme was assumed to replicate the same vaccine antigen coverage including the use of a ‘booster’ dose at 18 months. Vaccination coverage rates were taken as the average of those achieved in Chile between 2006 and 2015 [29].

Adverse event rates, associated with vaccination, were taken from previously published studies [30, 31] and included: seizures (with or without fever) and hypotonia-hyporesponsiveness syndrome after wP vaccines, edema, vomiting, anorexia, pain and redness or irritation of the skin. Full details of the values used can be found in Table 1. For seizures and other neurological effects such as hypotonic-hyporesponsiveness, data were taken from two studies which estimated seizures (with or without fever) and hypotonia syndrome; hypotonic- hyporesponsiveness episodes after wP vaccines was estimated at 1 case/1750 vaccinated i.e. 0.57 cases/1000 doses [32] and 0.12 cases/1000 doses with the aP vaccines [31].

**Table 1:** Adverse event rates for vaccines containing whole-cell pertussis and acellular pertussis components [30]

Adverse Event	1st Dose		2nd Dose		3rd Dose	
	Whole-cell vaccine (%)	Acellular vaccine (%)	Whole-cell vaccine (%)	Acellular vaccine (%)	Whole-cell vaccine (%)	Acellular vaccine (%)
Fever between 100.1°-101 °F (37.8–38.38 °C)	24.3	2.3	28.8	12.8	27.8	15.2
Fever between 101.1°-102 °F (38.39–38.89 °C)	3.0	0.8	3.9	0.8	7.3	2.3
Fever > 102 °F (> 38.9 °C)	0	0	1.4	0.8	2.6	0
Skin reddening (1-20 mm)	40.8	13.5	41.6	24.1	44.4	25.8
Skin reddening (> 20 mm)	8.6	0.8	6.1	1.5	3.2	3.0
Edema (1-20 mm)	23.2	7.5	26.6	16.5	30.1	14.4
Edema (> 20 mm)	16.5	0.8	9.5	0.8	5.6	3.8
Moderate pain	17.6	2.3	12.6	3.0	12.0	3.8
Severe pain	9.7	0	6.1	0	3.8	0
Moderate irritation	16.8	3.8	16.5	6.0	12.6	4.5
Severe irritation	3.8	0.8	7.0	0.8	4.7	0.8
Use of anti-pyretics	60.5	35.3	59.8	35.3	61.4	33.3
Fatigue	43.5	28.6	31.0	14.3	24.6	14.4
Reduced appetite	19.5	7.5	16.5	4.5	14.3	12.1
Vomiting	7.0	3.0	4.5	1.5	5.3	3.8

It was assumed that some of the more serious adverse events result in costs being incurred, for example through medical consultations, absenteeism and medication. Those events which were deemed to result in medical treatment were severe injection site edema > 20 mm; severe pain; severe irritation due to agitation or persistent acute crying; and drowsiness. Based upon expert opinion, we assumed that 20% would be the appropriate consultation rate. For those suffering from some more serious neurological manifestations (e.g. convulsions with or without fever, hypotonia-hyporesponse syndrome) it was assumed that all cases consulted were hospitalized for 1.5 days, and had a single post-discharge outpatient consultation.

Cost data was taken from a variety of sources including PAHO, previous vaccine studies and national estimates and included: antipyretics use, patient transfers/travel expenses, medical consultation, hospitalization, lost work time/days for parental care, time to administer the vaccine dose [33], cost of vaccine distribution, cost of the cold chain, costs for programmatic errors, costs of OPV, IPV, hexavalent and pentavalent vaccines, cost for the purchase of the hexavalent vaccine in the private sector. Details of the costs included can be found in Table 2.

**Table 2:** Costs included

Healthcare resource	Value (US$)	Source
Outpatient clinic	13.97	Arancel MAI 2018 MINSAL, Chile [34]: https://www.fonasa.cl/sites/fonasa/prestadores/normativa/aranceles#modalidad-de-atencion-institucional%2D%2Dmai-
Pediatric hospitalization (day)	556	Arancel MAI 2018 MINSAL, Chile [35]: https://www.fonasa.cl/sites/fonasa/prestadores/normativa/aranceles#modalidad-de-atencion-institucional--mai-
Anti-pyretic (Paracetamol 100 mg/mL)	6.89	CENABAST, Chile [36]: https://www.cenabast.cl/compras-cenabast-vigentes
Travel to hospital (2 journeys per visit)	1.11	Assumption: It was assumed that the transfer to ambulatory as well as to the emergency and hospitalization, required 2 transfers. The reference was transfer by bus, which costs 740 Chilean pesos, with an exchange rate to 666.26 pesos per US$, corresponds to a cost of US$ 1.11.
Loss of working time to attend outpatients/hospital	32.99 (per day. Assume 1 day per OP visit and 1.5 days per hospitalization)	Assumption: The average value of a working day corresponds to 21,977.26 Chilean pesos, equivalent to US$ 32.99. It was assumed that the loss would be 1 day in case of transfer to outpatient clinic and 1.5 days in the case of hospitalization.
Price of hexavalent (DTaP-IPV-HepB-Hib) vaccine	19.80	PAHO [37]**:**https://www.paho.org/hq/index.php?option=com_docman&view=download&category_slug=vacunas-9980&alias=38125-fondo-rotatorio-precios-vacunas-2017-125&Itemid=270&lang=es
Price of IPV in pre-filled syringe	5.3	Sanofi Pasteur and PAHO [37]: https://www.paho.org/hq/index.php?option=com_docman&view=download&category_slug=vacunas-9980&alias=38125-fondo-rotatorio-precios-vacunas-2017-125&Itemid=270&lang=es
Price of IPV plus cost of syringe	2.1	Price of vial (US$ 1.9) + syringe, PAHO [37]: https://www.paho.org/hq/index.php?option=com_docman&view=download&category_slug=vacunas-9980&alias=38125-fondo-rotatorio-precios-vacunas-2017-125&Itemid=270&lang=es
Price of IPV where supply difficulties require two different presentations to be used	3.6	Average between pre-filled syringe and vial, PAHO [37]: https://www.paho.org/hq/index.php?option=com_docman&view=download&category_slug=vacunas-9980&alias=38125-fondo-rotatorio-precios-vacunas-2017-125&Itemid=270&lang=es
Price of the bivalent oral polio vaccine (10-dose bottle)	0.32 (allowing for wastage)	PAHO [37]: https://www.paho.org/hq/index.php?option=com_docman&view=download&category_slug=vacunas-9980&alias=38125-fondo-rotatorio-precios-vacunas-2017-125&Itemid=270&lang=es
Price of the pentavalent (DTwP-Hib-HepB) vaccine (per dose)	2.85	Last adjudicated bidder for the pentavalent vaccine: GSK at US$ 1898 x dose. http://www.mercadopublico.cl/Procurement/Modules/RFB/DetailsAcquisition.aspx?qs=K4WQ9V4g07z52TulmJTwrg==
Administration of vaccine (**12** mins)	5.69 (Nursing assistant/hr)	Assumption: Includes: undressing the child, preparing the vaccine, applying, and registering in all documents and in the Nominal Registration System. It was assumed that the salary of a Nursing Assistant (who administers the vaccine at the vaccination center) would be 500,000 Chilean pesos, corresponding to 2727 Chilean pesos per day for 22 days per month from Monday to Friday. If the working day was 6 h, then the value of nurses time for the hour would be 3788 Chilean pesos, US$ 5.69)
Additional distribution costs of pentavalent (DTwP-Hib-HepB) plus polio vaccine	0.15	Assumption: A higher cost was assumed for distribution of vaccines in the pentavalent arm simultaneously with the polio vaccine. The cost of distribution per dose of vaccine was US$ 0.15. This cost was provided Sanofi Pasteur Chile from internal data.
Delivery costs of vaccine	3120 (per quarter)	Assumption: According to the size of the boxes, 9259 doses would be required to complete 1 cubic meter. Each maintenance panel was 1 cubic meter. The total doses would equal 104 panels, but an adequate stock per quarter was assumed with quarterly deliveries. The panel requirement was 26 per quarter (each month of the quarter) and with a cost of US$ 40 per panel per month, makes a total quarterly cost of US$ 3120. This cost was provided Sanofi Pasteur Chile from internal data.
Level of errors in non-hexavalent vaccine program associated with simultaneous vaccination requirements^a^	1%	Assumption: These errors could be due to the lack of inputs and the parents’ discomfort in front of the number of applications on the same day, with the refusal to accept it. This percentage was the result of consulting vaccination centers.
Private purchase of hexavalent (DTaP-IPV-HepB-Hib) vaccine	70.92	Based on sales in 2016 in Chile, 3.1% of the eligible population purchase the vaccine privately and thus would save the healthcare system US$ 2,194,580

^a^For example, administration of all required vaccines at any scheduled visit may not be strictly according to the national schedule due to limits in stock availability or parental decision, which necessitates additional visits to receive all required vaccines (with their associated costs)

All costs are in 2017 prices. Prices were converted from Chilean pesos into US$ using an exchange rate of $Ch 666.26 = US$1 (April 2017) [38].

Four scenarios were modelled (Table 3); all were conducted from a social perspective. In Scenario 1, the cost of the scheme with hexavalent vaccine was estimated according to its reference price in the Revolving Fund of PAHO for 2017 and compared with the cost of the 2016 plan, which implies the expenditure of pentavalent vaccine (reference price of the bidding in place), plus spending on polio vaccine according to its average price in the Revolving Fund and the additional costs involved in the simultaneous scheme with the pentavalent vaccine. In this scenario, the price of the polio vaccine was the average of the prices of 1 dose of IPV and 3 doses of OPV.

**Table 3:**
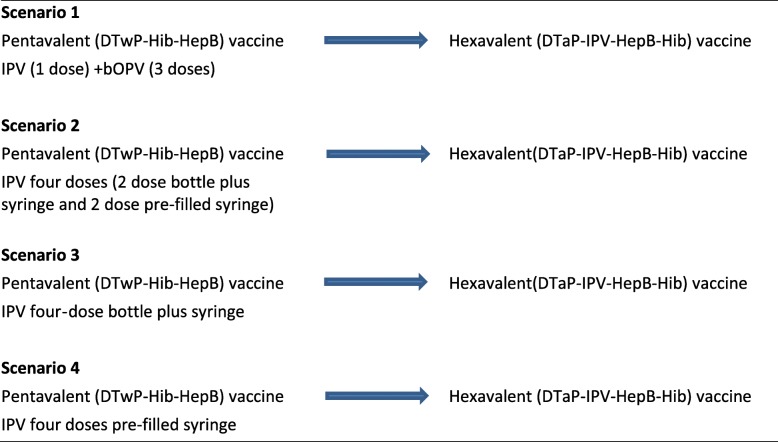
Summary of the switch from vaccination schemes based on the pentavalent vaccine with whole-cell pertussis component (wP) plus various combinations/presentations of IPV or OPV vaccines assessed in scenarios 1 to 4 to the hexavalent vaccine with acellular pertussis component (aP) and IPV

*bOPV* bivalent oral polio vaccine, *IPV* inactivated polio vaccine

Scenario 2 compared the current pentavalent vaccination plan and an additional 4-dose full IPV scheme versus vaccinating with hexavalent vaccine. The cost of the program with hexavalent vaccine was estimated based on its reference price in the Revolving Fund of PAHO of 2017 and compared with the cost of the current plan modified with the inclusion of a 4-dose full IPV scheme, which includes expenditure on the pentavalent vaccine (reference price of the tender in place), plus the average price of IPV in the PAHO Revolving Fund for 2017 for a 4-dose full IPV scheme and the additional costs which assumes the simultaneous scheme with the pentavalent vaccine. In this scenario, the price of IPV was the average of the prices of two different vaccines presentations in the Revolving Fund since there is a real possibility that the distribution of this vaccine through the Revolving Fund may vary due to supply issues.

Scenario 3 was similar to Scenario 2 above, but used the lowest reported price for IPV (US$ 2.10) reported in the PAHO revolving fund. Scenario 4, was also based on Scenario 2 above, but used the highest reported price for IPV (US$ 5.10) reported in the PAHO revolving fund.

## Results

The population of Chile was found to be relatively stable in the period 2010–2017 (Table 4). We used the data from the last full recorded year (2016) with a cohort of children aged < 1 year (249,552) and aged under 2 years (249,221) in this analysis.

**Table 4:** Population of Chile (projection of the 2 cohorts, 2007–2017)

Age	Year										
	2007	2008	2009	2010	2011	2012	2013	2014	2015	2016	2017
Total	16,504,869	16,686,853	16,876,767	17,066,142	17,255,527	17,444,799	17,631,579	17,819,054	18,006,407	18,191,884	18,373,917
0	235,457	242,535	248,363	250,453	248,203	244,493	243,846	246,478	248,803	249,552	248,757
1	231,072	235,687	242,775	248,628	250,790	248,599	244,906	244,260	246,895	249,221	249,979

From a societal perspective, the costs associated with the management of adverse events with the 2016 vaccination scheme (4 doses pentavalent vaccine + 1 dose IPV and 3 doses OPV) are US$ 8.84 million (Table 5) and those for the current vaccines used, US$ 3.86 million (Table 6). Thus, the overall costs associated with 2016 vaccination scheme are US$ 12.70 million. In comparison, the costs associated with the 4-dose scheme with a hexavalent vaccine (based upon the PAHO reference price) would be US$ 19.76 million (Table 6). Therefore, the cost of switching to the hexavalent vaccine would be an additional US$ 6.45 million (Scenario 1). The results for the other three scenarios are also shown in Table 6. The costs of switching to the hexavalent scheme would range from additional US$ 2.62 million to US$ 6.45 million compared with the current vaccination scheme according to the scenario considered.

**Table 5:** Summary of the additional costs involved in the use of vaccines containing whole-cell pertussis component (societal perspective)

Variable	First dose	Second dose	Third dose	Fourth dose	Total
HCP visits, hospitalizations and healthcare system costs	788,905	740,377	674,001	664,723	2,868,006
Pocket expenses, private vaccine payment	548,645	548,645	548,645	548,645	2,194,580
Transportations costs for adverse events	46,080	40,182	21,006	20,749	128,016
Absenteeism for parents for adverse events	686,022	598,436	317,221	304,666	1,906,345
Administration time for additional vaccine	269,567	269,567	266,730	263,542	1,069,406
Distribution costs of additional vaccine	35,561	35,561	35,187	34,766	141,075
Programmatic errors due to simultaneous vaccination^a^	129,767	129,767	129,767	129,595	518,896
Cold chain costs for additional vaccine^b^	3120	3120	3120	3120	12,480
**Total**	**2,507,667**	**2,365,655**	**1,995,667**	**1,969,806**	**8,838,805**

^a^For example, administration of all required vaccines at any scheduled visit may not be strictly according to the national schedule due to limits in stock availability or parental decision, which necessitates additional visits to receive all required vaccines (with their associated costs)

^b^Based on assumption of the volume of both schemes; considering that the size of vaccination cohort would be same for the hexavalent, as well as the pentavalent plus polio vaccine programs, then there would be a space reduction of 50% (1 vaccine versus 2 vaccines). The calculation of the cost of the square meter (cold chain pallet) was based on the additional square meters required for pentavalent plus polio vaccine program according to the Chilean cohort

*HCP* Health Care Provider

**Table 6:** Costs distribution according to scenario 1 to 4 from the societal perspective

	Unitary cost (US$)	Amount	Total (US$)
Scenario 1			
Hexavalent (DTaP-IPV-HepB-Hib) vaccine	19.80	997,877	19,757,965
Pentavalent (DTwP-Hib-HepB) vaccine	2.85	966,933	2,754,539
Average IPV (1) + bOPV (3) = 3.6 + (0.32*3) = 4.5	1.14	966,933	1,102,304
Difference of schemes	15.81	966,933	15,288,431
Additional costs of the simultaneous scheme			8,838,805
Cost to society			6,449,627
Scenario 2			
Hexavalent (DTaP-IPV-HepB-Hib) vaccine	19.80	997,877	19,757,965
Pentavalent (DTwP-Hib-HepB) vaccine	2.85	966,933	2,754,539
IPV four doses- average = 3.60	3.6	966,933	3,480,959
Difference of schemes	13.35	966,933	12,909,776
Additional costs of the simultaneous scheme			8,838,805
Cost to society			4,070,971
Scenario 3			
Hexavalent (DTaP-IPV-HepB-Hib) vaccine	19.80	997,877	19,757,965
Pentavalent (DTwP-Hib-HepB) vaccine	2.85	966,933	2,754,539
IPV four-dose bottle plus syringe = 2.10	2.10	966,933	2,030,559
Difference of schemes	14.85	966,933	14,360,176
Additional costs of the simultaneous scheme			8,838,805
Cost to society			5,521,371
Scenario 4			
Hexavalent (DTaP-IPV-HepB-Hib) vaccine	19.80	997,877	19,757,965
Pentavalent (DTwP-Hib-HepB) vaccine	2.85	966,933	2,754,539
IPV four doses- pre-filled syringe = 5.10	5.10	966,933	4,931,358
Difference of schemes	11.85	997,877	11,459,377
Additional costs of the simultaneous scheme			8,838,805
Cost to society			2,620,572

*bOPV* bivalent oral polio vaccine, *IPV* inactivated polio vaccine

The difference of 30,944 doses in the amount of hexavalent and the other vaccines is based on 7736 children in the private market receiving four doses of Hexavalent (3 + 1 scheme), and was assumed that the ministry of health would reimburse the vaccine for the whole cohort.

In Scenario 4, the switch to the hexavalent scheme would cost US$ 2.62 million.

## Discussion

In this study, we estimated and compared the difference in costs generated by two schemes with similar health outcomes but differences in the costs and safety, which were largely associated with logistics and adverse events management. The four scenarios analyzed considered two different approaches towards polio immunization, moving from a representation of Chile’s 2016 approach (1 dose of inactivated polio vaccine, followed by 3 doses of OPV) towards the GPEI objective in which OPV should be suspended as soon as possible, which means that the complete IPV scheme (i.e. all doses used are IPV) would become the preferred option. Several countries in Latin America have already expressed interest in a complete IPV scheme, similar to that introduced in Uruguay in 2012, but the supply of IPV has not been adequate or sufficiently reliable to fully comply with that policy. Based upon the PAHO reference prices, use of the hexavalent vaccine implies a greater expense versus simultaneous vaccination using the current pentavalent plus polio vaccines. However, the existence of more adverse events in the current scheme associated with wP compared to the aP formulation of the hexavalent scheme minimizes the cost difference between the two schemes.

There have been relatively few published assessments of the economic impact of hexavalent vaccines. A study in France (where the hepatitis B vaccine coverage was low) of a hexavalent combination vaccine, found that the public price associated with a break-even point would be €53.77. The annual additional reimbursed cost of protecting an infant against the risk of hepatitis B was €28.20 per child, or about €21 million for an annual cohort of 760,000 births (total cost, €35 million) [39]. Whilst there are problems with direct comparisons between France and Chile, due to differences in the healthcare systems and relative GDP of the two countries, the use of a hexavalent vaccine in our hypothetical model does produce similar increase in overall costs when factoring in the lower population covered in Chile.

Our findings are supported by a previous study conducted in Latin America. An earlier study in Mexico examined the impact of moving from OPV to IPV vaccination schedule. The authors found that changing from the existing OPV-based routine schedule and intensive supplementary activities to a sequential IPV-OPV routine schedule using a pentavalent and IPV/OPV vaccines would save US$ 14.52 per vaccinated child, and changing to a full IPV routine schedule would save US$ 9.41 per vaccinated child [40]. This latter figure is consistent with the outcomes reported in Scenario 4 of our analysis using the lower price for the hexavalent vaccine.

A similar cost-minimization analysis has been performed for hexavalent vaccine introduction in South Africa, which analyzed replacing aP pentavalent vaccine and Hep B by the hexavalent combination. The authors concluded that implementing a hexavalent vaccine in South Africa was highly recommended, because it reduces healthcare provider costs by simplifying logistics and delivery infrastructure: reduced clinic visits, vaccination errors, number of injections and side effects, which can be expected to translate to better acceptability, convenience and increased compliance [41].

The strengths of our analysis are that we utilized nationally available data, or taken from peer-reviewed scientific publications. Our analysis also produced results which are consistent with other previous analyses reported. One of the possible weaknesses of our analysis is that it relies on the assumption that the hexavalent vaccine has similar efficacy to the currently used vaccines. If this assumption were subsequently shown to be inaccurate, then the approach we took here (cost-minimization) would no longer be appropriate.

Contradictory evidence exists on the aP vs. wP benefits. On one side, concerns about the immunity conferred by aP vaccines not being as complete or as long lasting as that with wP vaccines [42], and it is also possible that the immunity elicited by wP vaccines may better protects against colonization and transmission than that elicited by aP vaccines [43, 44]. On the other side, some studies documented that the switch to aP vaccines reduced all-cause hospitalization in infants [45], and that this would expected to reduce global costs.

Our analysis may have underestimated the benefits of switching to a new hexavalent scheme since the reduction in immunizations required, might well be valued by the parents involved, leading to increased coverage and/or improved timeliness of infants immunization, which isn’t captured in our analysis [19–22].

## Conclusions

The introduction of a hexavalent (DTaP-IPV-HepB-Hib) vaccine into the immunization schedule in Chile would lead to additional acquisition costs, which would be partially offset by improved logistics, and a reduction in adverse events associated with the 2016 vaccines. This analysis provides decision makers with additional information on the potential impact of changes to the vaccination schedule caused by technical innovations, whilst reflecting the likely supply of alternative vaccines.

## Data Availability

All data generated or analysed during this study are included in this published article [and its supplementary information files].

## Abbreviations

aP: Acellular pertussis vaccine component
DP: Diphtheria and pertussis
DTP: Diphtheria, tetanus and pertussis
GPEI: Global Polio Endgame Initiative
HepB: Hepatitis B vaccine component
Hib: *Haemophilus influenzae* type b
IPV: Inactivated polio vaccine
OPV: Oral polio vaccine
PAHO: Pan American Health Organization
wP: Whole-cell pertussis vaccine component
WHO-SAGE: World Health Organization, Strategic Advisory Group of Experts
